# Bi-allelic truncating variants in *CASP2* underlie a neurodevelopmental disorder with lissencephaly

**DOI:** 10.1038/s41431-023-01461-2

**Published:** 2023-10-26

**Authors:** Eyyup Uctepe, Barbara Vona, Fatma Nisa Esen, F. Mujgan Sonmez, Thomas Smol, Sait Tümer, Hanifenur Mancılar, Dilan Ece Geylan Durgun, Odile Boute, Meysam Moghbeli, Ehsan Ghayoor Karimiani, Narges Hashemi, Behnoosh Bakhshoodeh, Hyung Goo Kim, Reza Maroofian, Ahmet Yesilyurt

**Affiliations:** 1 Acibadem Ankara Tissue Typing Laboratory, Ankara, Türkiye; 2 Institute of Human Genetics, University Medical Center Göttingen, Heinrich-Düker-Weg 12, 37073 Göttingen, Germany; 3 Institute for Auditory Neuroscience and InnerEarLab, University Medical Center Göttingen, Robert-Koch-Str. 40, 37075 Göttingen, Germany; 4 Acibadem Labgen Genetic Diagnosis Center, Istanbul, Türkiye; 5 Department of Child Neurology, Faculty of Medicine, Retired lecturer, Karadeniz Technical University, Trabzon, Türkiye; 6 Private Office, Ankara, Türkiye; 7 Institut de Génétique Médicale, Université de Lille, ULR7364 RADEME, CHU Lille, F-59000 Lille, France; 8 Ultramar Medical Imaging Center, Ankara, Türkiye; 9 Clinique de Génétique, Université de Lille, ULR7364 RADEME, CHU Lille, F-59000 Lille, France; 10 Department of Medical Genetics and Molecular Medicine, School of Medicine, Mashhad University of Medical Sciences, Mashhad, Iran; 11 Molecular and Clinical Sciences Institute, St. George’s, University of London, Cranmer Terrace, London, SW17 0RE UK; 12 Department of Medical Genetics, Next Generation Genetic Polyclinic, Mashhad, Iran; 13 Department of Pediatrics, School of Medicine, Mashhad University of Medical Sciences, Mashhad, Iran; 14 Mashhad University of Medical Sciences, Mashhad, Iran; 15 Neurological Disorders Research Center, Qatar Biomedical Research Institute, Hamad Bin Khalifa University, Doha, Qatar; 16 College of Health and Life Sciences, Hamad Bin Khalifa University, Doha, Qatar; 17 Department of Neuromuscular Disorders, UCL Queen Square Institute of Neurology, University College London, London, UK; 18 Acibadem Maslak Hospital, Istanbul, Türkiye

**Keywords:** Neurodevelopmental disorders, Disease genetics, Genetics research, Disease genetics, Genetic testing

## Abstract

Lissencephaly (LIS) is a malformation of cortical development due to deficient neuronal migration and abnormal formation of cerebral convolutions or gyri. Thirty-one LIS-associated genes have been previously described. Recently, biallelic pathogenic variants in *CRADD* and *PIDD1*, have associated with LIS impacting the previously established role of the PIDDosome in activating caspase-2. In this report, we describe biallelic truncating variants in *CASP2*, another subunit of PIDDosome complex. Seven patients from five independent families presenting with a neurodevelopmental phenotype were identified through GeneMatcher-facilitated international collaborations. Exome sequencing analysis was carried out and revealed two distinct novel homozygous (NM_032982.4:c.1156delT (p.Tyr386ThrfsTer25), and c.1174 C > T (p.Gln392Ter)) and compound heterozygous variants (c.[130 C > T];[876 + 1 G > T] p.[Arg44Ter];[?]) in *CASP2* segregating within the families in a manner compatible with an autosomal recessive pattern. RNA studies of the c.876 + 1 G > T variant indicated usage of two cryptic splice donor sites, each introducing a premature stop codon. All patients from whom brain MRIs were available had a typical fronto-temporal LIS and pachygyria, remarkably resembling the *CRADD* and *PIDD1*-related neuroimaging findings. Other findings included developmental delay, attention deficit hyperactivity disorder, hypotonia, seizure, poor social skills, and autistic traits. In summary, we present patients with CASP2-related ID, anterior-predominant LIS, and pachygyria similar to previously reported patients with *CRADD* and *PIDD1*-related disorders, expanding the genetic spectrum of LIS and lending support that each component of the PIDDosome complex is critical for normal development of the human cerebral cortex and brain function.

## Introduction

Lissencephaly (LIS) is a rare neurological disorder occurring due to an abnormal development of the cerebral cortex. This abnormality is caused by a deficient neuronal migration leading to abnormal formation of cerebral convolutions or gyri [1, 2]. LIS spectrum disorder includes agyria, pachygyria and subcortical band heterotopia [3]. Comorbidity of LIS includes intellectual disability (ID), developmental delay (DD), early hypotonia with subsequent spastic quadriplegia, and seizures [4].

There are 21 patterns of LIS spectrum, including partial or diffuse agyria-pachygyria, cerebellar dysplasia/hypoplasia, dysmorphic basal ganglia, thin or absent corpus callosum, abnormalities of the hippocampus and brainstem, posterior or anterior dominance of agyria, and pachygyria with mild cortical thickening [1, 3]. To date, 31 LIS-associated genes have been reported [3], including *CRADD* encoding CASP2 and RIPK1 domain containing Adapter with Death Domain, and *PIDD1* encoding P53-Induced Death Domain containing protein 1, both of which are components of the PIDDosome complex [5]. In this complex, CRADD functions as a dual adapter protein for activation of caspase-2 (CASP2). It contains a death domain that mediates interaction with *PIDD1*, and a caspase recruiting domain (CARD) domain, which mediates interaction with caspase-2 [6].

In 2016, Di Donato et al. showed *CRADD* biallelic loss of function variants as causal for LIS with megalencephaly, ID, and defined hallmark features of CRADD-associated phenotypes that include fronto-temporal predominant pachygyria-agyria with mild cortical thickening (OMIM: 614499) [7]. Recently, homozygous pathogenic variants in *PIDD1* have been reported in patients with non-syndromic ID (OMIM: 619827) [8], several neuropsychiatric and behavioral abnormalities, and a “CRADD-like” anterior-predominant pachygyria pattern in neuroimaging [9]. However, to date, the *CASP2* gene has not been associated with a human phenotype (OMIM: 600639).

Here, we present seven patients with DD/ID. All patients from whom brain MRIs were available have anterior-predominant LIS and pachygyria associated with biallelic pathogenic variants in *CASP2*, a member of the PIDDosome genes. The clinical and neuroimaging findings of the patients are remarkably similar to those of patients with CRADD- and PIDD-associated disease, supporting previous evidence that the PIDDosome protein complex is essential for normal development of the human neocortex and normal cognitive functions.

## Method

**Fig. 1:**
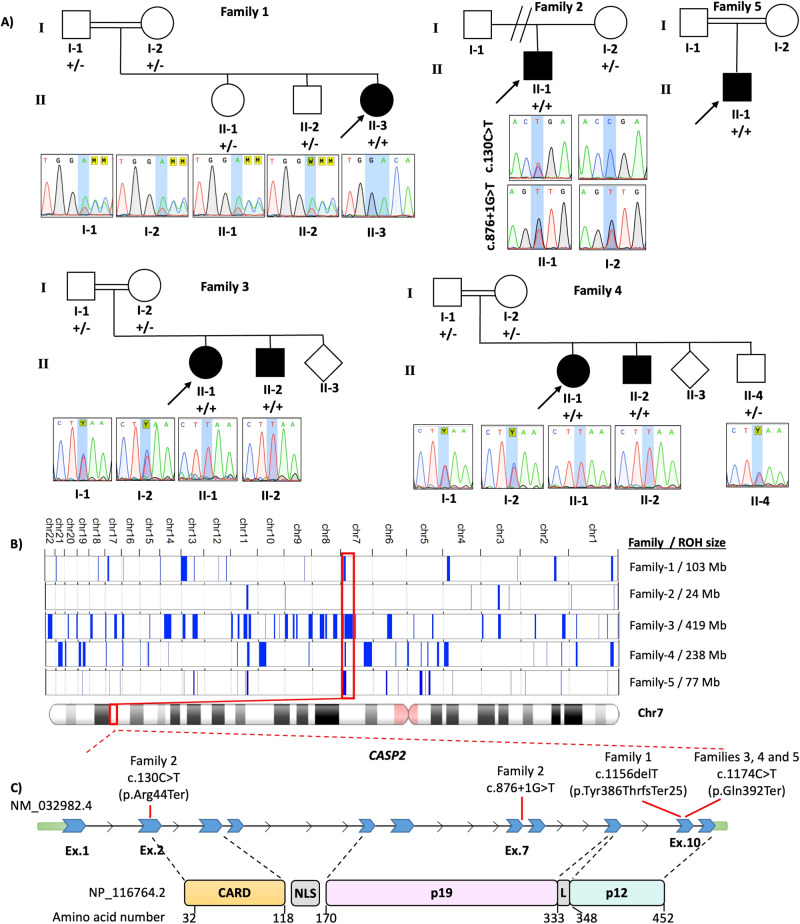
Pedigree and variants. **A** Pedigree and Sanger sequencing showing segregation of *CASP2* variants in the families. **B** Overview of the whole regions of homozygosity (ROH) in the exome of each patient from 5 families and total size of ROH for each proband [19]. The block of homozygosity surrounding the *CASP2* variant is indicated in red. **C** Schematic presentation of the pathogenic *CASP2* variants detected in the patients. CARD Caspase activation and recruitment domain of Caspase-2, NLS Nuclear localization signal, L Linker domain.

We evaluated four consanguineous and one nonconsanguineous families identified through data sharing with colleagues and using GeneMatcher [10] (Fig. 1A). Detailed clinical features as well as family history were obtained from families 1, 2 and 5, but were not available for families 3 and 4. Exome sequencing, as well as Sanger sequencing, were performed independently.

### Whole exome sequencing

Genomic DNA was extracted from proband blood samples using the QiaAmp system (Qiagen, Hilden, Germany). Exome sequencing was performed with the Twist Comprehensive Exome kit (Twist Bioscience, San Francisco, CA, USA) in family 1, Illumina DNA Prep with Enrichment kit (Illumina, San Diego, CA, USA) for family 2 and Nextera Rapid Capture Exome kit (Illumina) for families 3, 4 and 5. Bioinformatic analysis of raw data was performed with Varsome Clinical Software (Saphetor SA, Lausanne, Switzerland) after quality control and alignment to the reference genome GRCh37/hg19 for families 1 and 2 and was performed as previously described for families 3, 4 and 5 [11]. The fraction of targets covered at least 20X was 98.5 % and the average sequencing depth on target was 98 %. Variants were filtered according to ClinVar (pathogenic/likely pathogenic/conflicting interpretations of pathogenicity) [12], pathogenic/likely pathogenic variant classification by Varsome Clinical Software [13], Human Phenotype Ontology terms [14] associated with the clinical findings of the patient, and Genomics England Panels (Intellectual disability - microarray and sequencing, version 5.5) [15]. Computational assessment of splicing effects used SpliceSiteFinder-like, MaxEntScan, NNSplice, and GeneSplicer embedded in Alamut Visual Plus v1.6.1 (Sophia Genetics, Bidart, France) as well as SpliceAI Visual [16].

Due to parental consanguinity in families 1, 3 and 4 as well as lack of family history in all families, analysis of homozygous and compound heterozygous variants supporting a presumed autosomal recessive inheritance pattern were prioritized. All variants with a minor allele frequency less than 1% in gnomAD database were included. Variants were classified according to the Association for Clinical Genomic Science (ACGS) [17] and Clinical Genome Resource (ClinGen) Guidelines [18]. Deletion/duplication analysis was performed for patient 1 using the Varsome Clinical Software CNV Caller tool [13]. Region of homozygosity (ROH) analysis was performed using AutoMap [19].

### Segregation analysis

Genomic DNAs were isolated from whole blood samples of patients as well as available affected and unaffected family members. PCR amplification was carried out for the related exons of the *CASP2* gene using Veriti Thermal Cyclers (Applied Biosystems). The PCR products were purified and analyzed by Sanger sequencing following standard procedures. The sequence traces were aligned and analyzed by using SeqScape (Applied Biosystems).

### In vitro splice assay for functional analysis of the c.876 + 1 G > T variant

RNA studies were performed as previously described with modifications [20, 21]. Briefly, a 722 bp region spanning introns 7 to 9 were amplified from the patient DNA sample using restriction-site-containing primers (forward primer containing a *Xho*I restriction site: 5′- aattctcgagATCCAGGAGCTGTTTGCCTA-3′ and reverse primer with a *Bam*HI restriction site: 5′-attggatccCAATAGCACCTGAGAGGAGCA-3′). The PCR fragment was ligated between exons A and B of the linearized pSPL3-vector following restriction enzyme digestion. The recombinant vectors were transformed into DH5α competent cells (NEB 5-alpha, New England Biolabs, Frankfurt, Germany), plated and incubated overnight. Colony PCR with SD6 F (5′-TCTGAGTCACCTGGACAACC-3′) and the above reverse primer identified the wild-type and mutant-containing vectors that were sequence-verified and transfected into HEK 293 T cells (ATCC, Manassas, VA, USA) with a density of 2 × 10^5^ cells per mL. 2 µg of the respective pSPL3 vectors was transiently transfected using 6 µL of FuGENE 6 Transfection Reagent (Promega, Walldorf, Germany). An empty vector and transfection negative reactions were included as controls. The transfected cells were harvested after 24 h. Total RNA was prepared using miRNAeasy Mini Kit (Qiagen, Hilden, Germany). RNA was reverse transcribed using the High Capacity cDNA Reverse Transcription Kit (Applied Biosystems, Waltham, MA, USA) following the manufacturer’s protocols. cDNA was PCR amplified using vector-specific SD6 F and SA2 R (5′-ATCTCAGTGGTATTTGTGAGC-3′) primers. The amplified fragments were visualized on a 1% agarose gel and Sanger sequenced.

## Results

### Clinical and neuroradiological characteristics of the patients

#### Family 1

An 8-year-old female patient with Turkish ancestry was first admitted to the pediatric neurology clinic with complaints of delayed speech, attention deficit hyperactivity disorder, and inability of some motor activities at 5 years 10 months of age. She was born as the third child of a 37-year-old woman following uneventful gestation and delivery. Her parents were first-degree cousins (Fig. 1A). There was also a history of delayed speech in her father and her uncle.

The patient’s phenotype was characterized by normal early development, followed by delayed speech and language development. She especially had difficulties in learning grammatical rules such as usage of suffixes, and had particular difficulties with saying multi-syllable words. By the age of 7 years, there had been significant spontaneous improvement in her word count and language impairment.

Furthermore, she had attention deficit hyperactivity disorder and a history of Methylphenidate usage. On physical examination at 5 years and 10 months of age, her weight was 19 kg (25–50 centile), height was 114 cm (50–75 centile), and head circumference was 49.5 cm (10–25 centile). Physical and neurological examination was normal, except for hyperactivity and mild motor disability. She was unable to jump on one leg or from line to line. She has mild dysmorphic facial features such as synophrys, laterally flared thick eyebrows, upslanting palpebral fissures, a prominent nasal tip, a thin upper lip, a chin dimple, low-set, posteriorly rotated ears, and thin ear helix.

**Fig. 2:**
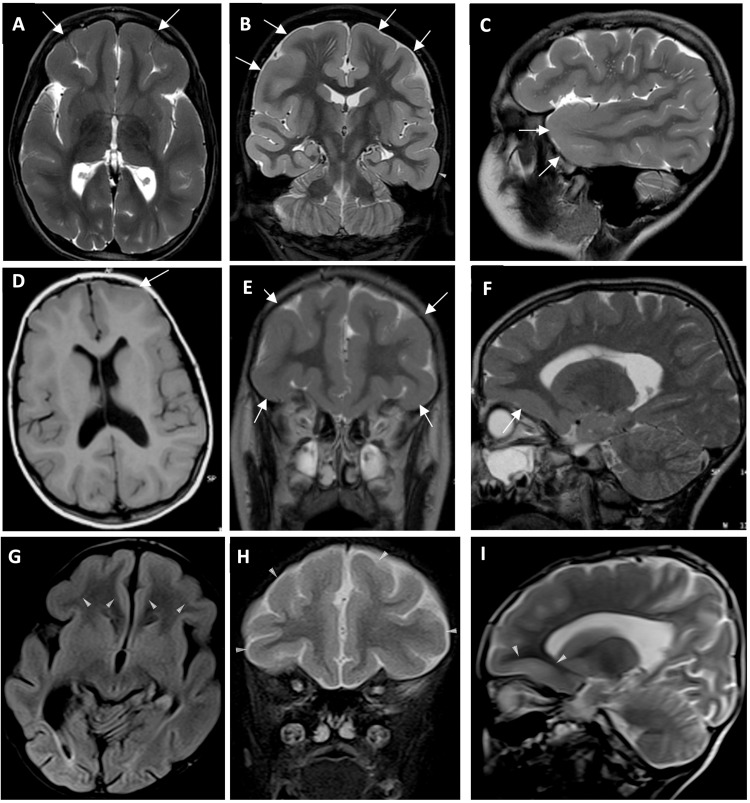
Brain MRI of the patients. **A**–**I** Brain MRI of Family 1, individual II-3, Family 2, individual II-1 and Family 5, individual II-1. **A** Axial **B** Coronal and **C** Sagittal T2-weighted image of Family 1, individual II-3, showing fronto-temporal pachygyria and mild cortical thickening (white arrows). **D** Axial T1-weighted **E** Coronal and **F** Sagittal T2-weighted image of Family 2, individual II-1, showing fronto-temporal pachygyria and mild cortical thickening (white arrows). **G** Axial T1-weighted **H** Coronal and **I** Sagittal T2-weighted image of Family 5, individual II-1, showing fronto-temporal pachygyria and mild cortical thickening (white arrows).

Her hemogram, biochemical investigations including liver and renal function testing, and CK were normal. Metabolic investigations, including ammonium, lactate, urine and blood amino acids, and urine organic acids, were also normal. EEG showed two different epileptiform activities originating from the right fronto-central region and fronto-temporal area. Brain MRI revealed bilateral pachygyria and mild cortical thickening in the fronto-temporal lobes. Perivascular spaces are prominent in the subcortical white matter of both frontal lobes (Fig. 2A, B, C). Sodium valproate therapy was started at 20 mg/kg/day. Currently, she is 8-years-old, and can speak fluently with multiple sentences, but has difficulties with using some conjunctions. She has learned to read but has difficulties in writing and in arithmetic. She experienced an improvement in hyperactivity to the extent that she no longer has difficulties in social interaction. The latest EEG of the patient revealed normal findings, and the dose of the sodium valproate therapy has been decreased.

#### Family 2

Family 2, individual II-1was a 26-year-old male with French ancestry who was born at term after an uneventful pregnancy. His parents were nonconsanguineous (Fig. 1A). At birth, his weight was 3700 g (54th centile), length was 52 cm (50th centile), and head circumference was 36 cm (51st centile). He walked at 12 months but showed regression after 24 months. At 30 months, pachygyria was identified in the context of atypical malaise with hypotonia. Subsequently, he presented with global DD without seizures. At 26 years old, he has speech delay, behavioral problems with intolerance to frustration, poor social skills, and autistic features. His neurological evaluation showed axial and peripheral hypotonias, and upper motor neuron signs, such as weakness, spasticity, and hyperreflexia (Table 1).

**Table 1:** Genetic and phenotypic features of patients with *CASP2* variants and literature review of patients with *CRADD* and *PIDD1*-associated diagnoses.

	Subjects with *CASP2* variants					Subjects with *PIDD1* variants					Subjects with *CRADD* variants				
	Family 1 (II-3)	Family 2 (II-1)	Family 3(II-1,II-2) and 4 (II-1,II-2)	Family 5 (II-1)	Total	Zaki et al. [9]	Sheikh et al. [27]	Harripaul et al. [8]	Hu et al. [32]	Total	Koprulu et al. [28]	Polla et al. [30]	Harel et al. 2016	Di Donato et al. [7] and Avela et al. [31]	Total
Self-reported ethnicity/country of origin	Turkish	French	Iran	Iran		Egyptian (3), Pakistani, Palestinian, Colombian	Indian	Pakistani (9)	Iran (5)		Pakistani	Finland	Jewish Bukharan	Turkish, Finland, Western Europe, Mennonite	
*CASP2* variant (NM_032982.4) or variant type (*PIDD1* and *CRADD*)	c.1156delT (p.Tyr386ThrfsTer25)	c.[130 C > T];[876 + 1 G > T]	c.1174 C > T (p.Gln392Ter)	c.1174 C > T (p.Gln392Ter)		Missense, nonsense, frameshift	Nonsense	Nonsense	Splice, missense		Missense	Missense	Frameshift	Missense, microdeletion	
Clinical features															
Intellectual disability / developmental delay	Mild ID	Global developmental delay, moderate ID	ID	Developmental delay	7/7	11/11	1/1	9/9	5/5	26/26	3/3	22/22	2/2	13/13	40/40
Autistic features	-	+	N/A	-	1/3	4/10	0/1	0/9	0/5	4/25	N/A	0/22	0/2	N/A	0/24
ADHD	+	-	N/A	-	1/3	6/10	1/1	2/9	0/5	9/25	3/3	7/22	0/2	N/A	10/27
Aggressive behavior	-	+	N/A	-	1/2	9/10	0/1	4/9	0/5	13/25	3/3	10/22	0/2	N/A	13/27
Other behaviors	-	Intolerance to frustration, poor social skills	N/A	-	1/3	5/11	1/1	3/9	3/5	12/26	3/3	4/13	0/2	N/A	7/18
Seizure	-	-	N/A	+	1/3	4/11	1/1	1/9	3/5	9/26	0/3	2/22	0/2	4/13	6/40
Neurological exam	-	Hypotonia, weakness, spasticity, and hyperreflexia	N/A	Hypotonia, poor vision, bilateral optic atrophy and abnormal uncoordinated gait	2/3	Hypotonia, strabismus, brisk DTR, mild gait instability, mild tremor	Normal	Bradychynesia, Strabismus	Normal	14/26	Strabismus	Strabismus	N/A	Normal	2/38
Dysmorphic features	Synophrys, laterally flared thick eyebrows, upslanting palpebral fissures, prominent nasal tip, thin upper lip, chin dimple, low-set, posteriorly rotated ears, and thin ear helix	-	N/A	-	1/3	11/11	1/1	1/9	2/5	15/26	3/3	2/22	0/2	0/13	5/40
Cranial MRI abnormality															
Lissencephaly (LIS)	Bilateral pachygyria and mild cortical thickening in the fronto-temporal lobes	Bilateral pachygyria and mild cortical thickening in the fronto-temporal lobes	N/A	Bilateral pachygyria and mild cortical thickening in the fronto-temporal lobes	3/3	10/10	1/1	9/9	N/A	20/20	3/3	17/17	3/3	13/13	36/36
Thin corpus callosum	-	-	N/A	-	0/3	5/10	1/1	1/9	N/A	7/20	0/3	0/17	0/3	2/13	2/36
Other brain MRI findings	Prominent subcortical perivascular space	-	N/A	Hydrocephaly	2/3	6/10	1/1	0/9	N/A	7/20	3/3	8/17	1/3	2/13	14/36

A bilateral pachygyria and mild cortical thickening in the fronto-temporal lobes was revealed from a brain MRI performed at 7 years old (Fig. 2D, E, F). EEG was normal at 6-years-old.

#### Families 3 and 4

Family 3, individual II-1 was a girl of Iranian ancestry who presented with ID. Her parents were first-degree cousins. Her brother (Family 3, individual II-2) also had ID (Fig. 1A). Family 4, individual II-1, a girl of Iranian ancestry, presented with ID. Her parents were first-degree cousins and her brother (Family 4, individual II-1) also had ID (Fig. 1A). More detailed clinical history and brain MRIs were not available for patients in the families 3 and 4.

#### Family 5

Family 5, individual II-1 was a 6-year-old from a distantly related Iranian parents who was born at term after an uneventful pregnancy (Fig. 1A). He had history of hypotonia, DD, hydrocephaly and seizures which was started at the age of 4 years old and respond well to medication bilateral optic atrophy as well as mildly abnormal uncoordinated gait (Table 1).

Brain MRI performed at the age of 5 years revealed a bilateral pachygyria and mild cortical thickening in the fronto-temporal lobes and hydrocephaly (Fig. 2G, H, I).

### Genetic findings

Exome datasets were evaluated to identify variants clinically relevant to the described phenotype. Following filtering for homozygous variants in Family 1, individual II-3, a homozygous frameshift candidate variant NM_032982.4:c.1156del in exon 10 out of 11 exons of *CASP2* residing in a 15 Mb ROH was identified (Fig. 1A, B). The c.1156delT variant is predicted to result in a premature stop codon p.(Tyr386ThrfsTer25) that would likely cause a truncated protein, with implications of impairing the function of the p12 domain of CASP2 protein. Sanger sequencing confirmed that none of the other healthy family members had this variant in a homozygous state (Fig. 1A).

**Fig. 3:**
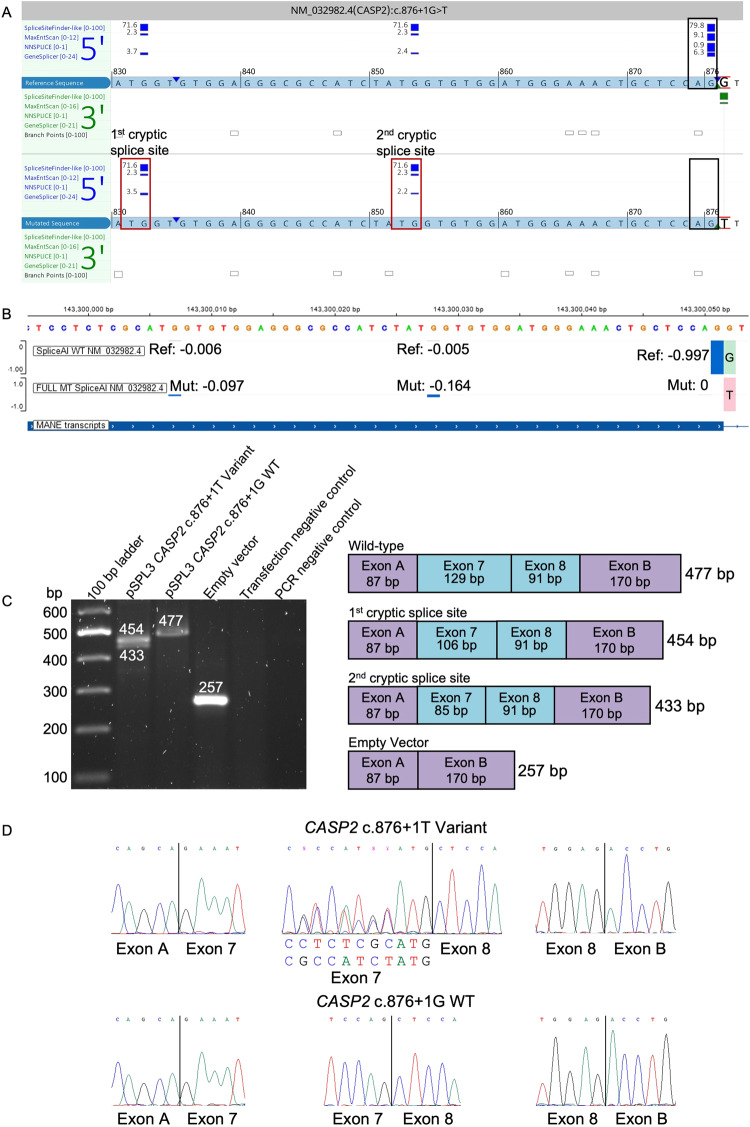
In silico prediction and RNA functional studies of the c.876 + 1 G > T variant. **A** In silico splice predictions of the wild-type (top track, red G) and c.876 + 1 G > T variant (bottom track, red T) are boxed in black. Only scores that are predicted to change are shown. The cryptic splice donor sites that are predicted to be activated due to abolishment of the native splice site are marked with red boxes and labeled as first and second cryptic splice site. **B** SpliceAI Visual prediction at the splice donor region of exon 7. Green and red bars on the right of the exon 7 boundary show the wild-type (G, top track) and mutant base (T, bottom track), respectively. Blue bars show the splice donor sites [score range: −1 to 0]. Here, a score closer to −1 represents a strong splice score. The score must be ≤ −0.2 in order to reach threshold. The native splice donor site is shown on the right with a score of −0.997. Two cryptic splice sites, shown with smaller blue bars slightly increase in score compared to the wild-type track. Ref reference, Mut mutant, WT wild-type. **C** Gel electrophoresis of the RT-PCR of the *CASP2* c.876 + 1 T variant, wild-type c.876 + 1 G, and empty pSPL3-vector amplicons. Transfection negative and PCR negative controls performed as expected. The splice schematic corresponding to each band size is shown on the right. **D** Sanger sequencing of each cDNA exon-exon junction. The *CASP2* c.876 + 1 T variant is shown in the upper panel. The middle panel shows the bases that originate each from the first (upper sequence) and second (lower sequence) cryptic splice sites. The *CASP2* c.876 + 1 G wild-type sequence is shown in the lower panel.

A duo exome sequencing was performed on the 26-year-old Family 2, individual II-1, and his mother revealing compound heterozygous variants NM_032982.4:c.[130 C > T];[876 + 1 G > T] p.[Arg44Ter];[?] in *CASP2* (Fig. 1A). The c.876 + 1 G > T variant was maternally inherited. Paternal DNA was unavailable for segregation analysis. The c.130 C > T variant is predicted to result in a nonsense mutation: p.(Arg44Ter) with probable nonsense-mediated decay (NMD). The c.876 + 1 G > T variant is located in the canonical splicing donor site of exon 7. In silico splicing predictions (SpliceSiteFinder-like, MaxEntScan, NNSPLICE, GeneSplicer and SpliceAI) unanimously concluded that the variant abolished the consensus splice donor site [22]. Four of five splice prediction tools suggested a cryptic splice donor site is possible (Fig. 3A), although the predicted cryptic splice donor score from SpliceAI did not reach threshold, which is set to ≤ −0.2 (range: [−1,0], with −1 set at maximum score; scores are shown in Fig. 3B as −0.098 and −0.164). RNA studies of the c.876 + 1 G > T variant confirmed usage of two cryptic splice donor sites (Fig. 3C, D). The first cryptic donor site results in a deletion of 44 bp of exon 7 (r.833_876del, p.(Gly278AlafsTer9)) whereas the second cryptic donor site causes a deletion of 23 bp (r.856_876del, p.(Gly285AlafsTer9)). In both instances, a premature stop codon will occur, likely triggering NMD.

Patients in Families 3, 4 and 5 were identified with a homozygous nonsense putative disease-causing variant NM_032982.4:c.1174 C > T in exon 10, residing in a 29 Mb and 5.6 Mb ROH, respectively (Fig. 1A, B). This variant is located 54 bp from the 3′ end of the penultimate coding exon of the gene leading to a premature stop codon p.(Gln392Ter) that is in close proximity to the NMD boundary. Functional studies of the variant and studies of patient cells were not performed, and therefore it remains unclear whether the mutant transcript leads to NMD or produces a truncated protein that impairs the protein function. Segregation studies in families confirmed that only affected individuals with ID had the candidate variant in a homozygous state, while healthy family members had either heterozygous or wild-type alleles (Fig. 1A).

We carried out run of homozygosity analysis for patients [19]. Consanguineous families all had ROH at 7q34, with a 5.6 Mb overlapping region which spans *CASP2* (Fig. 1B). All four variants (c.130 C > T, c.876 + 1 G > T, c.1156del, and c.1174 C > T) are not annotated in gnomAD [23]. Only the c.130 C > T was observed once among 276,302 alleles in a heterozygous state in the deCAF database [24]. Moreover, no other candidate variants were identified in well-known LIS-related genes in the patients’ exome data (Table 1S).

## Discussion

*CASP2* encodes the Caspase-2 protein, which belongs to a family of proteases that mediate apoptosis. It is the most evolutionarily conserved caspase and is accepted as an initiator caspase based on its activation process [25]. Caspase-2 regulates diverse stress-induced signaling pathways, with genotoxic stress being the most well-known activator for the caspase-2 pathway [26]. It contains a CARD domain (Fig. 1C), which mediates interactions with CRADD to facilitate the formation of the multiprotein PIDDosome complex, composed of PIDD, CRADD, and caspase-2 (Fig. S1). Similarly, CRADD has a death domain, and its interaction with the death domain of PIDD1 activates caspase-2 [5].

In the past few years, biallelic pathogenic variants in *CRADD* and *PIDD1* have been associated with LIS, anterior-predominant pachygyria, and ID (Table 1) [7–9, 27–32]. This finding has drawn attention to the PIDDosome complex, suggesting additional biological functions aside from DNA-damage induced apoptosis. The regulatory link between these three components of the PIDDosome have been highlighted with functional evaluation of biallelic pathogenic variants in *CRADD* and *PIDD1*, which have been associated with a complete loss of Caspase-2 activity [6, 33, 34]. However, it has also been shown that Caspase-2 can be activated without the PIDDosome complex, implying that there may be alternative PIDD-independent pathways for Caspase-2 activation in mammals [35]. Therefore, the association of ID and LIS with biallelic *CASP2* variants in the present study reinforces the implication of all components of the PIDDosome complex in LIS.

The first report implicating *CRADD* in isolated LIS involved 13 patients with homozygous pathogenic variants in the death domain, causing reduced Caspase-2 mediated neuronal apoptosis without disrupting interactions with Caspase-2 or PIDD [7]. All patients shared isolated LIS without other congenital anomalies. In addition to LIS, mild to moderate ID, relative or absolute megalencephaly, and seizures were other phenotypic features in the patients (Table 1). The authors proposed that reduced apoptosis is a novel developmental mechanism for cortical malformations [7].

The phenotypic variability of *CRADD* variants has been confirmed through a case series of 22 Finish patients with a homozygous founder allele [30]. Early psychomotor development was considered normal in patients whose learning development was then marked by mild or moderate ID or global DD at the age of 4 years. The patients had delayed language development, and their speech improved spontaneously by school age, similar to Family 1, individual II-3 in the current study, whose language impairment also improved spontaneously by the same age period. The researchers suggested that the disease hallmark is fronto-temporal predominant pachygyria with mild cortical thickening. In this series, aggressive behavior was found in nearly half of the patients, EEG abnormalities in five patients, and megalencephaly in three patients (Table 1).

The first report implicating *PIDD1* in ID involved two unrelated Pakistani patients with homozygous nonsense pathogenic variants disrupting the death domain [8]. Sheikh et al. reported functional and cellular analysis of *PIDD1* pathogenic variants, including effects on autoprocessing, interactions with CRADD, and caspase-2 activity and linked them with lissencephaly for the first time [27]. Also, interestingly, they reported that *Pidd1* knockout mice showed no central nervous system (CNS) /behavioral phenotype, similar to *Casp2* knockout mouse. They proposed it might be due to very restricted *Pidd1* expression, and low degree of transcriptional overlap between *Pidd1, Cradd*, and *Casp2* in mice CNS cells in contrast to human CNS cells. They suggested that that further studies into other PIDDosome pathway components as disease genes for neurodevelopmental disorders because of the phenotypic commonalities observed in humans thus far.

Zaki et al. reported 11 patients with biallelic pathogenic variants in the *PIDD1* gene [9]. All patients exhibited DD, and variable degree of ID. Six patients had attention deficit/hyperactivity disorder. Additionally, mild and nonspecific dysmorphic features were reported in most patients. Brain MRIs of the patients revealed a spectrum of cerebral cortical anomalies, mainly consistent with anterior-predominant pachygyria (Table 1). The thickness of the cortex was reported to be above the normal limit of 4 mm for most cortical regions in the patients.

Thus, similar to biallelic *CRADD* and *PIDD1* loss of function, we observed a mild to moderate neurodevelopmental disorder associated with biallelic *CASP2* variants. The neurological patterns of these patients are remarkably similar to those of *CRADD*-related patients with LIS, suggesting dysregulation of the same pathways regardless the implicated gene [7, 9].

In current study, Family 5, individual II-1 has bilateral optic atrophy and concomitant hydrocephalus, although none of the other patients with detailed clinical data has optic atrophy. Severe visual problems and optic atrophy has been reported in untreated patients with hydrocephaly [36]. It is shown that inhibition of caspase-2 expression neuroprotective effects for retinal ganglion cells loss, and enhanced retinal ganglion cell survival in optic nerve damage [37–39]. So, we conclude that optic atrophy in Family 5, individual II-1 may be due more to secondary to hydrocephalus rather than having truncating *CASP2* variants.

Caspase-2 undergoes autocatalytic activation to remove the prodomain and linker region to generate a stable dimer consisting of the large subunit (p19) and the small subunit (p12) (Fig. 1C) [40]. This p19/p12 dimer self-associates to form the active caspase-2 [41]. The c.1156delT and c.1174 C > T variants reside in exon 10 out of 11 and is predicted to result in a premature stop codon and can lead to a truncated protein. This truncated protein may cause impairment in the p12 dimerization, and therefore caspase-2 activation.

The Human Protein Atlas demonstrated medium to high levels of CASP2 expression in the human brain (Fig. S2) [42]. Expression in the mouse brain was determined high at birth, with progressive decline during postnatal development [43]. Moreover, *CASP2* is transcribed in both the developing and the adult human brain (Fig. S3) [44]. Additionally, coexpression of PIDD1, CRADD, and CASP2 is very high in multiple human brain regions during both development and adulthood [45, 46]. Contrasting the detrimental loss of function effect in PIDDosome complex proteins in human brain morphology, *Casp2*^*-/-*^ mice did not show a significant CNS phenotype, implying that Caspase-2 activity may be less critical in organization of the mouse brain than it is in the human brain [47]. The exclusive role of Caspase-2 in brain development has not been elucidated until now. Our study supports the evidence that Caspase-2 plays an essential role both in the cortical development and cognitive function of the human brain similar to *CRADD* and *PIDD1*.

NMD surveillance following RNA biogenesis uses cap-binding protein and exon-junction complexes to distinguish between regular versus premature stop codons. The exon-junction complex is deposited upstream of spliced exon-exon junctions. The majority of exon-junction complexes require 20–24 nucleotides upstream of the exon-exon junction and serve as a landmark for splicing machinery [48]. NMD uses the cap-binding complex to identify the termination codon within the reading frame. Ribosomes remove exon-junction complexes and establish whether termination codons fall 50–55 nt upstream of an exon-exon junction that is an indication of premature termination and a signal for NMD [49]. Therefore, if the premature termination codon is at least 50–55 nt upstream of the 3′-exon-exon junction, through initiation of NMD, unfavorable transcripts are avoided via degradation [50]. NMD is likely to happen in both variants in individual II-1 in Family 2, c.130 C > T, p.(Arg44Ter), c.876 + 1 G > T, while likely not occurring in individual II-3 in Family 1 with the c.1156delT variant, as the frameshift and premature truncating codon preserves the original exon 10 length. The variant in the affected individuals from Families 3–5 occurs upstream of the 3′-most exon-exon junction by 53 nt, therefore making the consequence of this variant challenging to predict.

In summary, we present seven patients with *CASP2*-related DD/ID and all patients for whom brain MRIs were available have anterior-predominant LIS and pachygyria in neuroimaging, similar to previously reported *CRADD* and *PIDD1* patients. This finding supports previous evidence that PIDDosome complex proteins are critical for the normal development of the human cerebral cortex and cognitive function. Our data expand the genetic spectrum of LIS and indicate biallelic truncating variants in CASP2 underlies autosomal recessive LIS and DD/ID in patients.

### Supplementary information

Supplementary Figures and Table

## Data Availability

All data concerning this work is included in the manuscript and its supplement. All variants included in the manuscript have been uploaded to ClinVar (https://www.ncbi.nlm.nih.gov/clinvar/; submission VCV002499471.1, VCV002499469.1, VCV002499470.1). The datasets generated and/or analyzed during the current study are available from the corresponding author on reasonable request.
